# Neglected orbitozygomaticomaxillary fractures with complications: A case report

**DOI:** 10.1016/j.ijscr.2019.07.055

**Published:** 2019-07-26

**Authors:** Siti Isya Wahdini, Ishandono Dachlan, Rosadi Seswandhana, Magda Rosalina Hutagalung, Indri Lakhsmi Putri, Dwiki Afandy

**Affiliations:** a Division of Plastic, Aesthetic & Reconstructive Surgery, Department of Surgery, Faculty of Medicine, Public Health and Nursing, Universitas Gadjah Mada/Dr. Sardjito Hospital, Yogyakarta, Indonesia; b Department of Plastic, Aesthetic & Reconstructive Surgery, Faculty of Medicine, Universitas Airlangga/Dr. Soetomo Hospital, Surabaya, Indonesia; c Faculty of Medicine, Public Health and Nursing, Universitas Gadjah Mada, Yogyakarta, Indonesia

**Keywords:** Orbitozygomaticomaxillary fracture, Maxillofacial trauma, Surgical repair, Case report

## Abstract

**Introduction:**

Zygomatic complex fracture is one of the most frequently occuring injuries to facial structures and often involves damage to the surrounding structures, including the orbital floor and/or medial orbital wall. These complicated injuries can cause both serious opthalmic and aesthetic complcations that make reconstructions more difficult.

**Case presentation:**

A 22 year old male presented with facial asymmetry. On examination, there were neglected fractures of left orbitozygomaticomaxillary complex with various compications. We performed lateral orbitotomy, bone graft, medial and lateral cantopexy with 3D reconstruction models as a guidance. There were some appearance improvements including improved orbital dystopia and corrected facial asymmetry. However, his vision could not be corrected because the reconstruction was too late.

**Discussion:**

Several examinations before reconstruction of the injury has to be done, especially radiological examinations to decide proper diagnosis and reconstruction plans. Some complications may make difficulties in establishing precise time for surgery. Three-dimensional (3-D) reconstrucion model can help increase accuracy of the reconstruction but consume more time.

**Conclusion:**

Severe maxillofacial fracture should be treated immediately to avoid further complications. Multidisciplinary examinations can provide more accurate preliminary recommendations particularly when combine with properly calibrated CT scan imaging.

## Highlights

• Zygomatic complex fractures can involve surrounding structures and cause serious ophthalmic and aesthetic complications.
• Radiological investigations is very useful in diagnosing and planning the reconstruction surgery.
• The goal of fracture reconstructions is to restore the appearance and skeletal anatomy before injury instead of bone healing.
• Immediate reconstruction is recommended to prevent more complications.
• 3-D model reconstruction can provide better accuracy but takes longer time.

## 1 Introduction

Facial reconstruction surgery is common because the face is a prominent point of injury due to sports injuries and vehicular accidents. The cheek bones and eye sockets often need repair requiring extensive rebuilding [1]. Several cosmetic procedures have been developed and the most frequently occuring injury is the zygomatic complex (ZMC) fractures characterized by fracture (s) of the zygoma or adjacent bones, such as the maxilla, orbit, or temporal bone [2,3]. Those ZMC fractures often involve damage to the orbital floor and/or the medial orbital wall [4].

The orbital floor has a fragile structure, making it easily damaged in a craniomaxillofacial trauma. The area located in medial to the infraorbital groove and canal are main locations of sustained fractures. Sometimes, the fractures involve damage to medial orbital wall because of the reduced bone thickness in that area [5]. These fractures can cause serious ophthalmic and aesthetic complications. Serious ophthalmic complications include visual disturbance, diplopia, and enophthalmos, while facial asymmetry is an aesthetic complication results from the injuries [6]. These complications can give significant challenges to the plastic surgeon to repair.

This case report follows SCARE criteria [7].

## 2 Case presentation

**Fig. 1:**
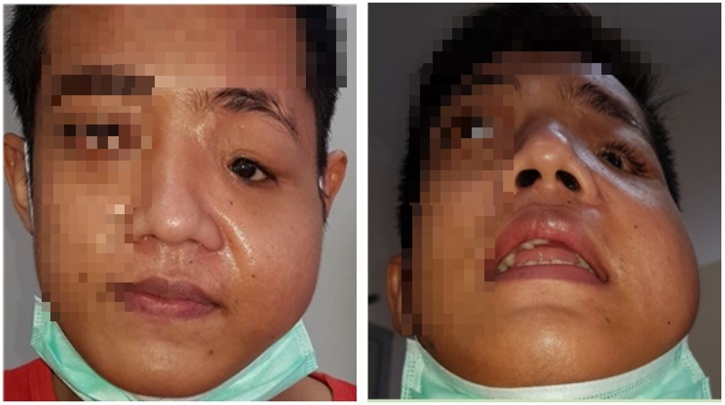
The patient’s physical appearance before reconstruction.

**Fig. 2:**
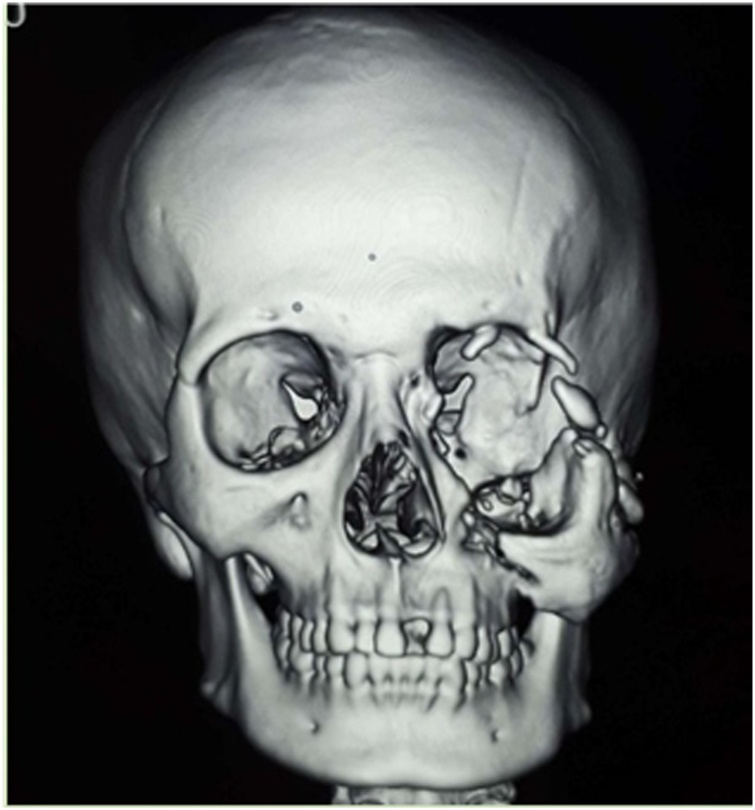
Three-dimensional CT-scan before reconstrucrion.

A 22 year old male presented to the Division of Plastic, Reconstruction, and Aesthetic Surgery, in the Department of Surgery of our institute with facial asymmetry. He was injuried in a traffic accident 2 years ago and had underwent interdental and intermaxillary wiring by a surgeon at a district hospital. The patient did not feel any improvements and then came to our institute. On the physical examination, there was maxilla deformity on the left side (Fig. 1). Opthalmology examination found light perception only in the left eye. This came along with orbital dystopia and obstruction of nasolacrimal duct. Then we performed 3-D CT scan and turned out that there was a fracture of left orbitozygomaticomaxillary complex. It showed shattered orbital plate and processus frontalis. Zygoma bone and maxilla bone also shifted to below of its proper position (Fig. 2).

**Fig. 3:**
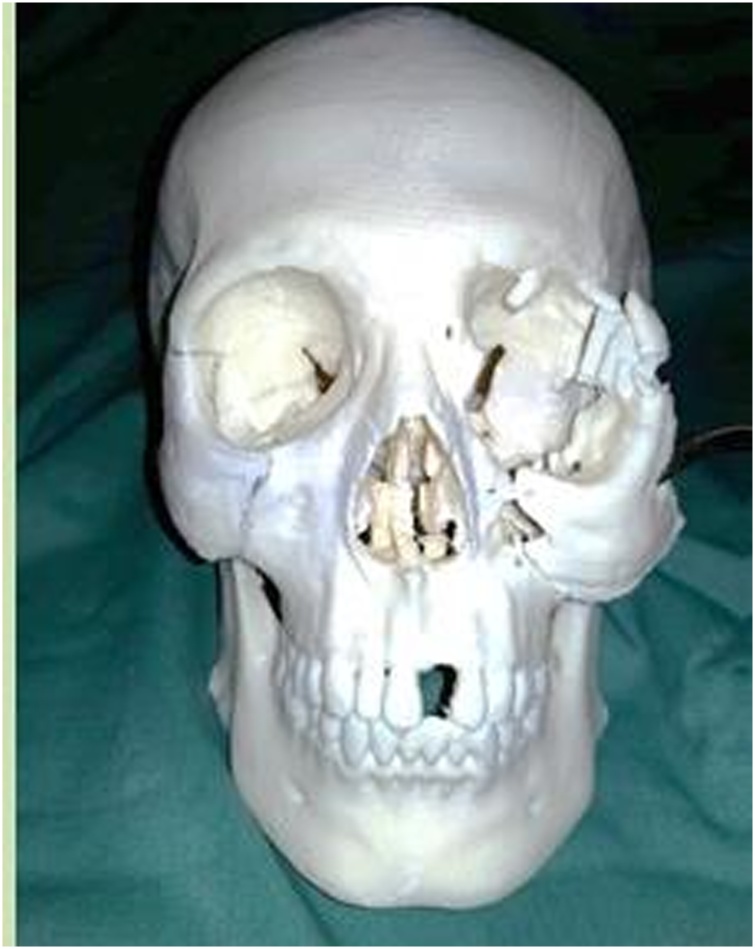
Three-dimensional (3-D) reconstructions model used as guidance during surgery.

**Fig. 4:**
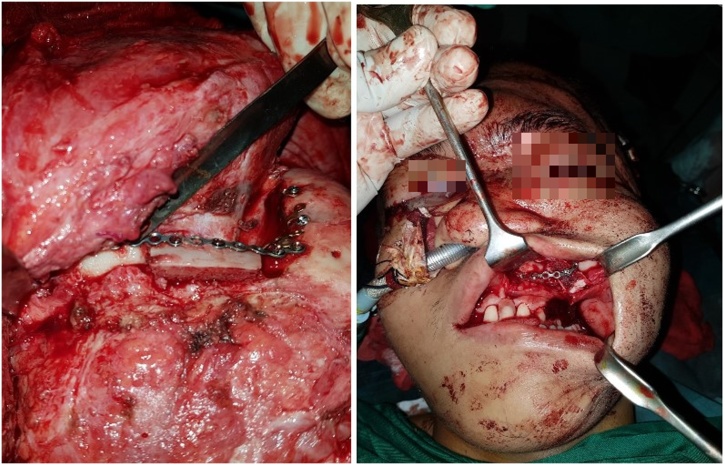
Bone graft installment in frontozygoma (left) and maxilla (right).

**Fig. 5:**
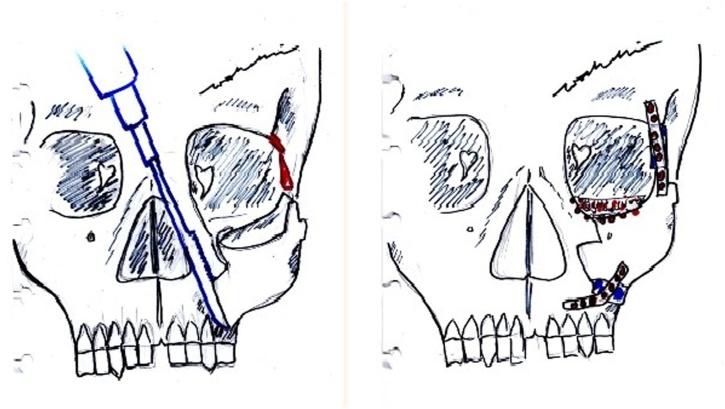
Schematic picture of our reconstruction approach (left) and the result of reconstruction (right). (*Courtesy of Siti Isya Wahdini*).

We did the reconstruction using bicoronal approach and 3D reconstruction models as a guidance for the surgical procedures (Fig. 3). We performed zygomaticomaxilla osteotomy and Tessier’s inferior orbital marginotomy. Bone graft was harvested from 8th rib bone with 6,5 cm total length. 3,5 cm was applied to frontozygoma and 3 cm to left maxilla (Fig. 4). The bone graft then was fixed with plate. Medial and lateral cantopexy were installed with screw. The schematic picture is showed in (Fig. 5).

**Fig. 6:**
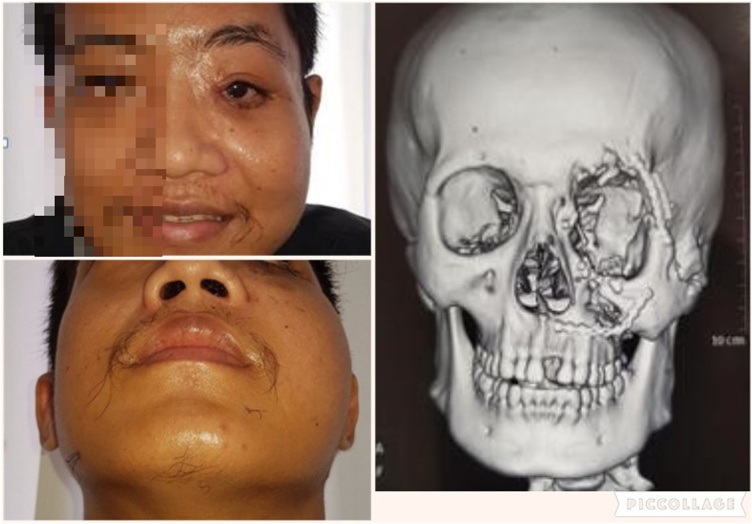
The patient’s physical appearance and three-dimensional CT-scan after reconstruction.

In the 4th month after operation the cantopexy was still broken causing sagging of the cheek skin to be prominent. The 2nd operation was performed to fix the sagging cheek by mini face lift and orbital dystopia by adding bone wax and orbital mesh, and also reconstruction of nasolacrimal duct. There were some appearance improvements including corrected orbital dystopia and improved facial asymmetry (Fig. 6). Also, he had no more epiphora. However, his vision could not be corrected because it was too late.

## 3 Discussion

Fractures of the orbitozygomaticomaxillary complex are involved in nearly 30% of all facial fractures [8], while orbital fractures represent over 40% of maxillofacial injuries, making them the most common trauma in the midface. Diagnosis of ZMC involves fracture of the lateral orbital wall and articulation of the zygoma with the greater wing of the sphenoid [9]. Manson et al. describe the injury around the zygomatic bone as low energy, middle energy, and high energy injuries [10]. In our report, the patient belongs to the high energy injury due to the comminution in the lateral orbit and telescoping of the zygomatic arch.

Healthcare providers who take care of these patients have to understand the related physical examinations, including opthalmology findings. Any misdiagnosis or incorrect reconstruction of the preinjury anatomy can cause post traumatic deformities of the orbit that can lead to serious complications, including enophthalmos, diplopia, and visual acuity disturbance [11]. Early diagnosis and management of any facial injury is essential to prevent late treatment and possible permanent disfigurement. Preoperative work on these patients has to be done properly including computed tomography (CT) scans [9]. Correct phantom calibration is essential for CT image readings and proper diagnosis.

A variety of defects in the orbital wall can be captured with CT imaging ranging from hairline fractures without displacement to full separation of orbital bones which can cause tissue prolapse in the eye socket and surrounding orbitomaxillary structures including the cheek bone and detached facial muscles. During repair of the injury to the zygoma, early exploration of the internal orbit can provide a foundation for later cosmetic surgery by rebuilding the orbital floor [12]. In this patient, we report a succesful repair using a mesh floor to support the subsequent face lift and cosmetic tissue repair.

According to Nigel et al., orbital floor fractures are considered urgent with following complications: diplopia with restriction of gaze, entrapment of extraocular muscles, enophthalmos >2 mm, fracture that involved over half the orbital floor and the medial wall, and “trapdoor” orbital floor fractures in children [8]. A systematic review by Dubois et al. identified some patients who developed challenging enopthalmos or persistent diplopia 2 weeks post injury. These complications create difficulties in early treatment recommendations particularly when patients have sustained only small damage to the eye and may not need surgery [13].

Opthalmologic findings and CT scan imaging can contribute significantly to recommendations for surgery and may need further evaluations from other disciplines such as neurosurgery for possible traumatic brain injury [5,13]. The goal of fracture reconstructions at the orbital area is different with fractures at long bones. They aim to restore the appearance and skeletal anatomy before injury instead of bone healing [14]. The time of surgery is still controversial. Burstine criteria is used often to decide the best time of surgery but must be revisited. Dubois et al. discussed recent studies of the effects of surgical timing on post-traumatic orbital reconstruction outcomes, which provide criteria for immediate repair and augmented guidelines to determine the ideal time for non-immediate surgery [13].

High energy injuries also often require extensive osteotomy, repositioning, and cranial bone graft replacement of displaced, partially resorbed, and comminuted bone. Extended open reduction and rigid fixation techniques in treating high-energy fractures are highly required, since the soft tissue deforming forces overwhelm the limited fixation of comminuted fractures, particularly fixation provided by interfragment wiring. A common combination of procedures is osteotomy and repositioning of the zygomatic arch to decrease facial width and custom onlay grafting of the malar prominence [14]. In the first treatment of our patient, he only underwent interdental wiring-intermaxillary wiring. It was an inadequate treatment that made him present to our hospital with complications. Then we performed lateral orbitotomy, bone graft, medial and lateral cantopexy with 3-D reconstruction model as a guidance for the surgeries.

Orbital reconstruction in orbital fracture treatment using implant materials is effective, but it is difficult to assess its accuracy during surgery. Therefore, a three-dimensional (3-D) reconstruction model was created as guidance during surgery. The use of 3-D models has significantly aided the surgical planning and treatment outcome of both pathologic and traumatic maxillofacial conditions. The main goal is restoration of the original orbital volume and prevention of long term complications [6,15]. Although this method easily reproduces accurate orbital positioning and shape, the prolonged time required to create and prepare these custom made pre-bending plate systems make it inapplicable during emergencies [13].

## 4 Conclusion

We succesfully treated a neglected case of orbitozygomaticomaxillary fracture with 2 years onset that never reported in any literature before. Bone healing process normally involves callus resorption by osteoclast and formation of lamellar bone by osteoblast. There is no any healing of injured bone in the result of imaging in this patient. Our approachment to this case give a satisfying outcome despite of inevitable vision loss. When complex and severe maxillofacial fractures occured especially with high energy injury causing opthamology and aesthetic complications, these cases should be referred to a plastic surgeon immediately. Early reconstruction is recommended although its accuracy has to be performed carefully because the 3-D reconstruction model takes longer time. Multidisciplinary examinations can provide more accurate preliminary recommendations particularly when combine with properly calibrated CT scan imaging.

## Sources of funding

This research did not receive any specific grant from funding agencies in the public, commercial, or not-for-profit sectors.

## Ethical approval

Ethical clearance is not needed in the ethics commission at our institution for a case report.

## Consent

Written informed consent was obtained from the patient for publication of this case report and accompanying images. A copy of the written consent is available for review by the Editor-in-Chief of this journal on request.

## Author’s contribution

SIW is the author conceived the study, and ID, RS, MRH, and ILP did the supervision. DA contributed in data collection.

## Registration of research studies

We use registry body from Clinicaltrials.gov with registration number NCT00583856.

## Guarantor

Siti Isya Wahdini.

## Provenance and peer review

Not commissioned, externally peer-reviewed.

## Declaration of Competing Interest

The authors declare that they have no competing interests.
